# Ca^2+^ waves in astrocytes: computational modeling and experimental data

**DOI:** 10.3389/fncel.2025.1536096

**Published:** 2025-03-28

**Authors:** Rosa Musotto, Ulderico Wanderlingh, Giovanni Pioggia

**Affiliations:** ^1^National Research Council, IRIB-CNR, Institute for Biomedical Research and Innovation, Messina, Italy; ^2^Department of Mathematical and Computer Sciences, Physical Sciences and Earth Sciences, University of Messina, Messina, Italy

**Keywords:** model, calcium wave, astrocytes, simulation, experimental data

## Abstract

This paper examines different computational models for Calcium wave propagation in astrocytes. Through a comparative analysis of models by Goldbeter, De Young-Keizer, Atri, Li-Rinzel, and De Pittà and of experimental data, the study highlights the model contributions for the understanding of Calcium dynamics. Tracing the evolution from simple to complex models, this work emphasizes the importance of integrating experimental data in order to further refine these models. The results allow to improve our understanding of the physiological functions of astrocytes, suggesting the importance of more accurate astrocyte models.

## Introduction

The field of neuroscience, and more specifically computational neuroscience, has in recent decades focused almost exclusively on the study and modeling of neuronal components and dynamics at both the cellular and network levels, almost completely neglecting the role of astrocytes except for their metabolic and homeostatic activity. Recent studies have shown that astrocyte Ca^2+^ variation is associated with the modulation of neuronal signaling through the uptake and release of neurotransmitters (Haydon and Carmignoto, 2006; Volterra and Meldolesi, 2005; Khakh and Mccarthy, 2015; Pasti et al., 1997; Fiacco and Mccarthy, 2006; Kofuji and Araque, 2021; Semyanov et al., 2020; Verkhratsky and Nedergaard, 2018; Khakh and Sofroniew, 2015). A growing body of research demonstrates that astrocytes are more than merely passive read-out units (Temburni and Jacob, 2001); rather, they play a significant role in controlling the activity of neuronal synapses (Fellin et al., 2006; Perea et al., 2009; Clarke and Barres, 2013). Astrocytes have a sort of chemical excitability based on variations in intracellular Calcium concentration, despite not being electrically excitable cells that is, they cannot produce action potentials. Astrocytes control the number of neurotransmitters in the synaptic cleft by regulating intracellular and intercellular Calcium dynamics; thereby controlling the synaptic signal current between two neurons. It is now known that astrocyte Ca^2+^ signaling is essential for proper functioning of neuronal activity and dysfunction of astrocyte dynamics is implicated in the onset of neurodegeneration (Kang et al., 2005; Nadkarni and Jung, 2003; Tian et al., 2005; Verkhratsky et al., 2010; Eddleston and Mucke, 1993; Rappold and Tieu, 2010; Eid et al., 2008; Madinier et al., 2013; Mitroshina et al., 2022; Jiang et al., 2024).

The discovery that astrocytes are responsible for neuronal activity has led to the creation of various mathematical and computational models for simulating astrocyte dynamics. Of these, those relating to the modulation of intracellular Ca^2+^ waves occupy particular importance due to their importance in cell communication. Research on glia entered a new era with the fundamental discovery in the 1980s that astrocytes express a wide range of receptors for neurotransmitters. Subsequent research has shown that the release of neurotransmitters during synaptic activity can activate these receptors and cause an increase in Ca^2+^ in astrocytes. In turn, this mechanism can cause the release of gliotransmitters such as glutamate, ATP and D-serine, which are capable of activating neuronal receptors, thus modifying the electrical excitability of neurons and synaptic transmission, triggering intercellular communication between astrocytes and neurons (Araque et al., 1999; Fellin et al., 2004; Schipke and Kettenmann, 2004; Jourdain et al., 2007). Thanks to these findings, the theory of “tripartite synapses” was developed, which considers astrocytes as the third component of the signal integration unit (Volterra et al., 2002). Recently, much research has been conducted on the mechanism of chemical transmitter release from astrocytes. Of all the gliotransmitters, glutamate has undoubtedly attracted the most attention due to the fundamental discovery by Anne Cornell-Bell and colleagues that glutamate evokes increased Calcium concentrations in astrocytes (Cornell-Bell et al., 1990).

Various studies have been done to confirm that astrocytes possess specific receptors for glutamate on the outer surface of the plasma membrane (mGluRs) (Anderson and Swanson, 2000; Backus et al., 1989; Condorelli et al., 1997). The function of glial mGluRs is still almost unknown, on the contrary, there is much evidence on the role of ionotropic glutamate receptors in glial cells (Dantoni et al., 2008; Verkhratsky and Steinhäuser, 2000; Kondoh et al., 2001; Seifert and Steinhäuser, 2001). Astrocytes release glutamate, which diffuses into the extra synaptic space and binds to metabotropic glutamate receptors (mGluRs) or NMDA receptors (NMDARs) of neighboring presynaptic terminals in turn, they may respond to the glutamate released at the synaptic level with an increase in intracellular Ca^2+^ that may trigger the release of further glutamate by astrocytes (Malarkey and Parpura, 2008; Skowrońska et al., 2019; Santello and Volterra, 2009; Montana et al., 2006).

Modeling and theoretical study of Ca^2+^ dynamics involving the IP_3_ receptor channel are the main topics of the review. It also provides a synopsis of the experimental results.

The models presented in this review are united by the fact that the dynamics of IP3 and the compartmental changes of Ca^2+^ are integrated in a set of ordinary differential equations. System parameters have a sensitive effect on the propagation of released Ca^2+^. Therefore, instead of reviewing the results of each study, we will present the ideas and techniques employed.

### Section of models

#### The Goldbeter model

**Figure 1:**
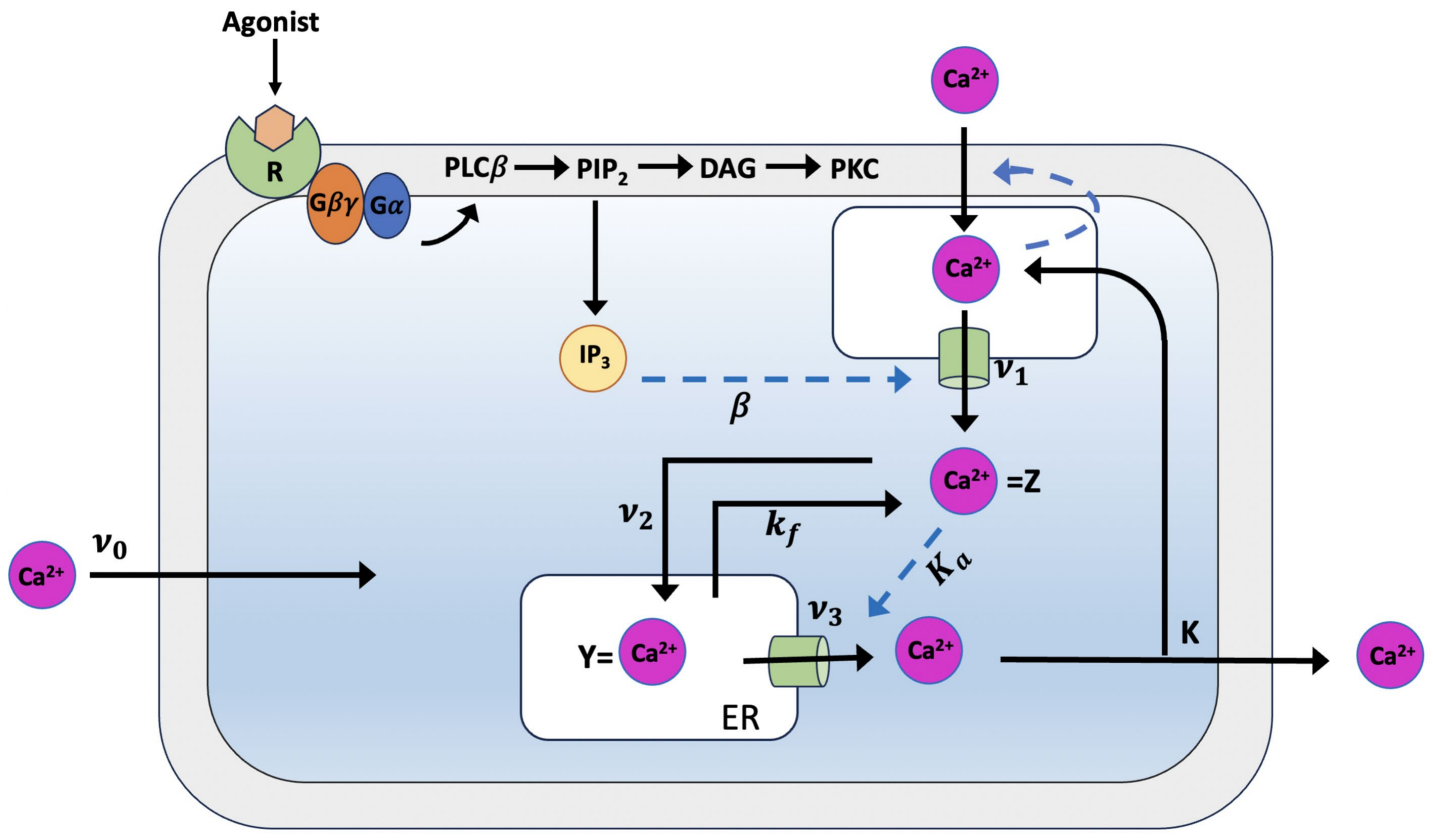
Illustration of the production mechanism of Ca^2+^ oscillations according to Goldbeter model, which is based on Ca^2+^ release induced by intracellular stores. Ca^2+^ release is modulated by IP_3_ from an IP_3_-sensitive store located in the cytosol $\left(\nu_{1}\right)\text{,}$ which also indirectly controls the influx of external Ca^2+^ into this store. In the model, $\nu_{1}$ determines the constant Ca^2+^ flux in the cytosol, which is controlled by each level of InsP3. *Z*, the cytosolic Ca^2+^ concentration, passes from a phase of low concentration, during which priming Ca^2+^ is transferred $\left(\nu_{2}\right)$ into the InsP3-insensitive pool, to a phase in which the Ca2+ stored in that pool (*Y*) is released into the cytosol $\left(\nu_{3}\right)\text{;}$ this phase is characterized by short peaks of Ca^2+^. The parameter $\nu_{0}$ refers to the influx of extracellular Ca^2+^ into the cytosol, k to the influx of cytosolic Ca^2+^ from the cell to the extracellular space and $k_{f}$ to the passive loss of *Y* in *Z* (see text for details).

Pioneering models for intracellular Ca^2+^ signaling include the Goldbeter et al. model (Santello and Volterra, 2009), which predicts the occurrence of periodic spikes of the ion in the absence of IP_3_ oscillations, indicating that repetitive Ca^2+^ spikes do not necessarily require a concomitant periodic change in IP_3_ and can be induced by external stimulation. The model assumes the existence of two distinct internal stores, one sensitive to IP_3_ and the other sensitive to Ca^2+^. The IP_3_ produced by agonist stimulation leads to a release of Ca^2+^ from the IP_3_-sensitive store via the IP_3_Rs. The released Ca^2+^ will stimulate a further release from the Ca^2+^ sensitive store (see Figure 1), which self-amplifies above a threshold value for cytosolic Ca^2+^ concentration (C), representing a model for Induced Calcium Release (CIRC). Depletion of the Ca^2+^-sensitive pool (C_ER_) limits the release. This model makes the critical assumption that the Ca^2+^ in the IP_3_-sensitive store remains constant as the extracellular medium rapidly replenishes it. The model lacks a mechanism for IP_3_-dependent Ca^2+^ inhibition. The two variables in the model are the concentration of free Ca^2+^ in the cytosol and in the IP3-insensitive repository (e.g., the endoplasmic reticulum or sarcoplasmic reticulum); these variables are denoted Z and Y, respectively. Assuming that buffering is linear with respect to Ca^2+^ concentration, the time evolution of the systems is governed by the two kinetic equations:


(1) $$\frac{dC}{dt}=\nu_{0}+\nu_{1}\beta -\nu_{2}+\nu_{3}+k_{f}Y-k_{Z}$$

(2) $$\frac{dC_{ER}}{dt}=\nu_{2}-\nu_{3}-k_{f}Y$$

In Equation 1, the ν_0_ parameter, which is assumed take constant, relates to the Ca^2+^ input from the extracellular medium into the cell; *k_Z_*, which is assumed to be linear, pertains the outflow of Ca^2+^ into outflow from the cell, which occurs even in the absence of external stimulation. *ν_1_β* denotes the InsPs-modulated release of Ca^2+^; *ν_2_* indicates the rate of ATP-driven pumping of Ca^2+^ from the cytosol into the InsP_3_-insensitive store, while *ν_3_* represents the rate of transport from this pool into the cytosol; finally, the term *k_f_Y* refers to a nonactivated transport of *C* into *C_ER_*.

When the cell receives an external signal, this triggers an increase in InsP_3_, which leads to a rise in the saturation function *β* and, subsequently, to an increase in cytosolic Ca^2+^.


(3) $$\nu_{2}=V_{M2}\frac{Z^{n}}{K_{2}^{n}+Z^{n}}\;\nu_{3}=V_{M3}\frac{Y^{m}}{K_{R}^{m}+Y^{m}}\cdot \frac{Z^{P}}{K_{A}^{P}+Z^{P}}$$

Were *V_M2_* and *V_M3_* denote, respectively, the maximum rates of Ca^2+^ pumping into and release from the intracellular store; these processes are described by Hill functions whose cooperativity coefficients are taken as *n* and *m*; *p* denotes the degree of cooperativity of the activation process; *K_2_*, *K_R_*, and *K_A_* are threshold constants for pumping, release, and activation.

The Goldbeter model assumes that two different types of pools are required for Ca^2+^ oscillations, some of which are sensitive to InsP_3_ and others with RyR and thus sensitive to Ca^2+^. Due to the InsP_3_R’s inherent sensitivity to both Ca^2+^ and InsP_3_, this proved unneeded. Subsequently, Dupont and Goldbeter formulated a version of the model that assumes the existence of a single pool in which Ca^2+^ and IP_3_ are co-agonists for the induction of Ca^2+^ release (Dupont and Goldbeter, 1993; Table 1).

**Table 1:** Parameters of the Goldbeter model (Goldbeter et al., 1990).

Parameters of Goldbeter model		
Parameter	Value	Description
ν_0_	1.0 μMs^−1^	Constant influx of Ca^2+^ in to the cell
ν_1_	7.3 μMs^−1^	InsPs-modulated release of Ca^2+^ from the InsP3-sensitive store
k	10.0 s^−1^	Constant efflux of Ca^2+^ in to the cell
k_f_	1.0 s^−1^	Rate constant measuring the passive, linear leak of cytosolic Ca^2+^into the extracellular medium
V_M2_	65.0 μMs^−1^	Maximum values of the pumping of Ca^2+^ into the InsP_3_-insensitive store
V_M3_	500.0 μMs^−1^	Maximum values of the release of Ca^2+^ into the InsP_3_-insensitive store
k_2_	1.0 μM	Threshold constants for Ca^2+^ pumping
k_R_	2.0 μM	Threshold constants for Ca^2+^ release
k_A_	0.9 μM	Threshold constants for Ca^2+^ activation
n	2	Hill coefficients characterizing these processes
m	2	Hill coefficients characterizing these processes
p	4	Hill coefficients characterizing these processes
β	30.1%	External stimulation

#### The De Young-Keizer model

**Figure 2:**
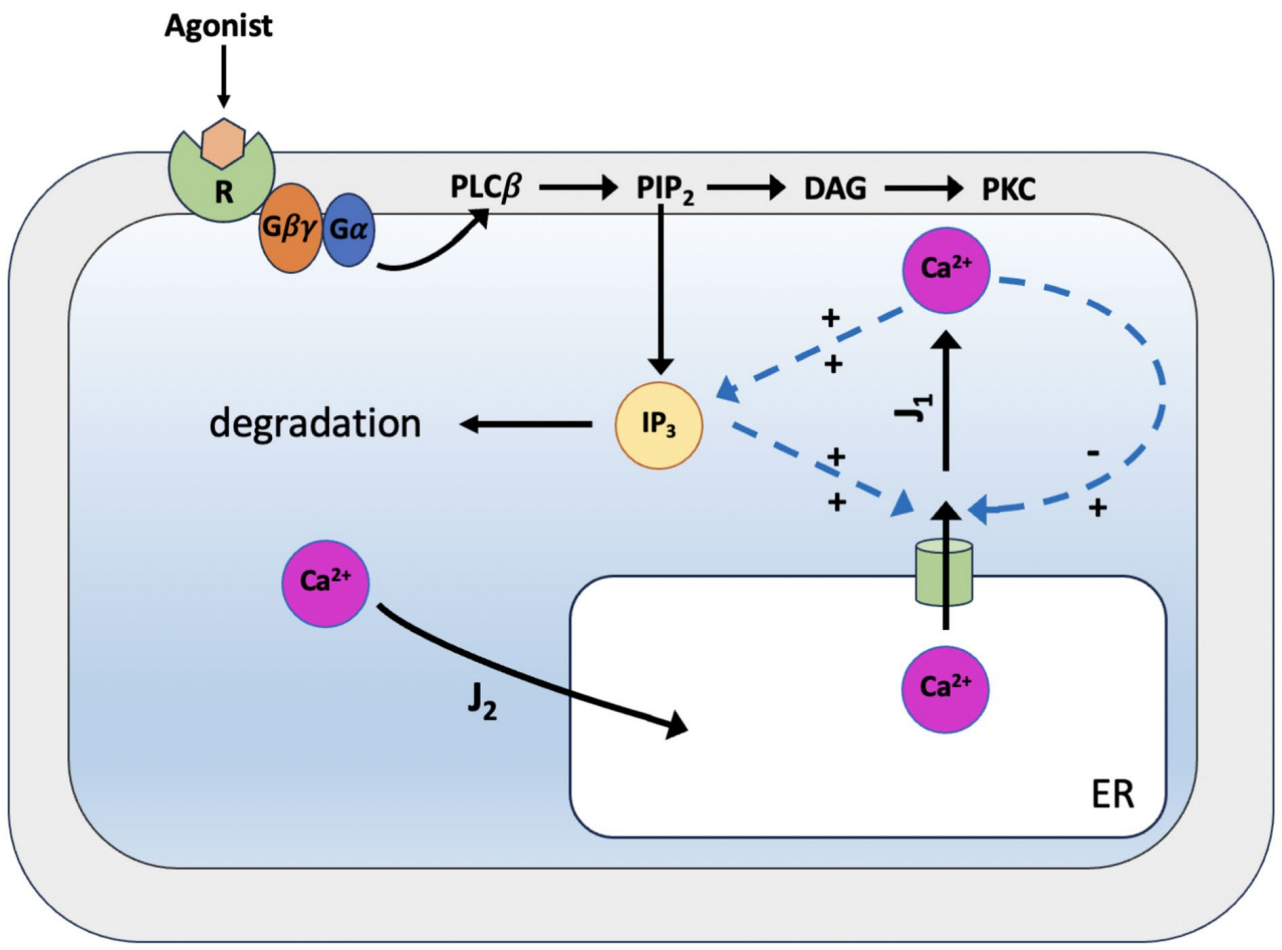
Scheme of the simplified De Young-Keizer kinetic model describing the properties of Ca^2+^ activation and inhibition by the inositol 1,4,5-trisphosphate (IP_3_) receptor in the endoplasmic reticulum. *J_1_* is the outward flux of Ca^2+^, and *J_2_* is the inward flux. *J_1_* has two components, the Ca^2+^ flux through the IP_3_ receptor/channel and a constant leak flux. *J_2_* represents the flux facilitated by the ATP-dependent Ca^2+^ pumps which actively transport Ca^2+^ from the cytosol back into the endoplasmic reticulum. The model incorporates the activity of Ca^2+^-ATPase, which is responsible for pumping Ca^2+^ back into the endoplasmic reticulum, and results in oscillations of cytoplasmic Ca^2+^ concentrations when the IP_3_ concentration is held constant. This occurs with only a single pool of Ca^2+^ available for release from the endoplasmic reticulum.

**Figure 3:**
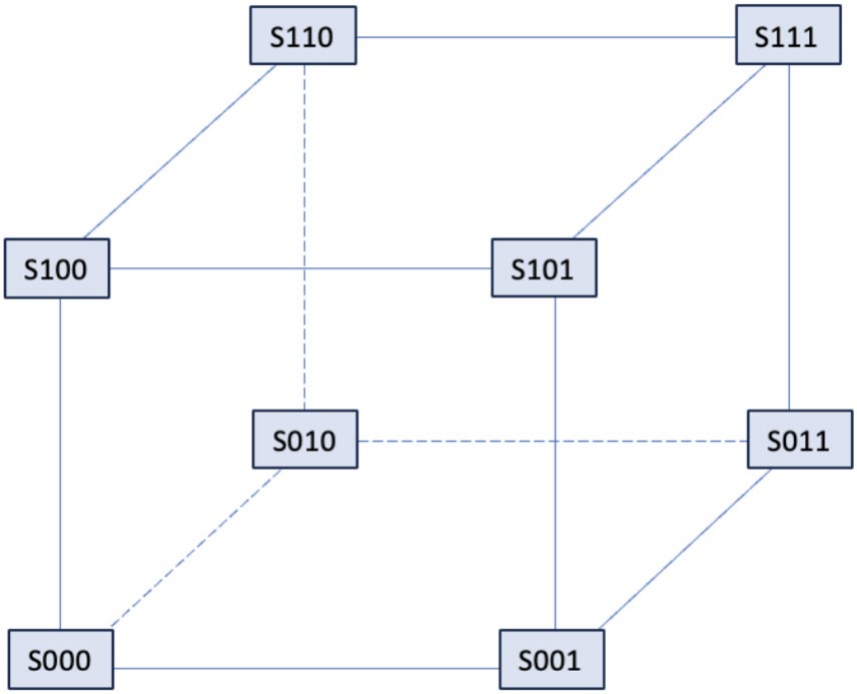
The IP3 receptor’s single unit’s binding mechanism. The probability that the component will be in one of the states [*i,j,k*], where *i, j,* and *k* can take the values 0 and 1, is shown by *S_ijk_*. The IP_3_ binding site’s condition is indicated by the first index, the activating Ca^2+^ binding site by the second, and the inhibitory Ca^2+^ binding site by the third. The corresponding binding site is unbound if an index is zero, and bound if an index is one.

**Figure 4:**
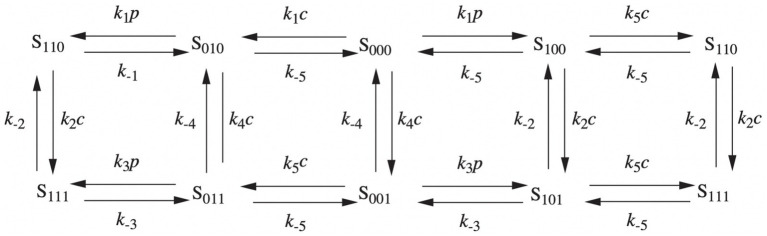
Binding diagram of De Young-Keizer IP_3_ pattern in two dimensions. *c* stands for Ca^2+^ and *p* for IP_3_.

In 1992, the De Young-Keizer model (Young et al., 1992) studied the properties of the IP_3_ receptor/ Ca^2+^ channel; in particular, it examined the biphasic response of the IP_3_ receptor/channel to cytosolic Ca^2+^ and how this could be sufficient to induce Ca^2+^ oscillations. The rate constants in the equations were fitted to the kinetic and equilibrium data and the model successfully reproduced a series of *in vivo* and *in vitro* experiments (Berridge and Irvine, 1989; Mouillac et al., 1990; Smrcka et al., 1991; Taylor and Exton, 1987). The model incorporates a positive Ca^2+^ feedback mechanism on IP_3_ production by phospholipase-C (PLC). It was noted that this enriches the properties of oscillations and leads to Ca^2+^ oscillations accompanied by IP_3_ oscillations (see Figure 2). They created a simplified model of the IP_3_ receptor/channel by assuming that Ca^2+^ conduction is mediated by three equivalent, independent subunits, all of which must be in a conducting state before the receptor allows Ca^2+^ to flow. There are three binding sites on each subunit, one for IP_3_, one for Ca^2+^ activation and one for Ca^2+^ inactivation. Consequently, each subunit can exist in eight states, with transitions controlled by first and second order rate constants for association and dissociation, respectively. Each state is labeled with *S_ijk_* the first index refers to the IP_3_ binding site, the second to the Ca^2+^ activation site and the third to the Ca^2+^ inactivation site; *i,j,k* take the value *0* or *1* depending on whether the binding site is unoccupied or occluded (see Figures 3, 4).

The 24 not-all-independent speed constants of the model were reduced to 10 constants, $k_{\pm }1,\ldots ,k_{\pm }5$ by introducing the following two assumptions:

i. the rate constants are independent of whether or not Ca^2+^ is bound to the activation site
ii. Ca^2+^ activation kinetics do not depend on IP_3_ or Ca^2+^ inactivation.

Since experimental data indicate that the receptor subunits act cooperatively, for the channel to be open and in conduction, all three subunits must be in the *S_110_* state (one bound to IP_3_ and one to activating Ca^2+^). The gives rise to seven differential equations for the receptor states. Although there are eight states, only seven are independent. As far as mass-action kinetics are concerned, the Ordinary Differential Equations (ODEs) for the receptor states have the present form:


(4) $$\begin{array}{l} \frac{dx_{000}}{dt}=\left(k_{-1}x_{100}-k_{1}px_{000}\right)+\left(k_{-4}x_{001}-k_{4}cx_{000}\right)+\\ \left(k_{-5}x_{010}-k_{5}cx_{000}\right) \end{array}$$

where *p* denotes [IP_3_] and *c* denotes [Ca^2+^].

The DeYoung and Keizer model consists of seven ODEs for receptor states with the following Equations 5–7 that describing the [Ca^2+^] handling of the IP_3_-sensitive Ca^2+^ pool and the IP_3_ production:


(5) $$\frac{\mathrm{dc}}{dt}=J_{1}-J_{2}$$

where *c* is the cytosolic free Ca^2+^ concentration, *J_1_* is the outward flux of Ca^2+^ and *J_2_* is the inward flux (see Figure 2).


(6) $$J_{1}=c_{1}\nu_{1}x_{110}^{3}\left(c_{ER}-c\right)+c_{1}\nu_{2}\left(c_{ER}-c\right)$$

(7) $$J_{2}=\frac{\nu_{3}c^{2}}{c^{2}+k_{3}^{2}}$$

*J_1_* has two components, the Ca^2+^ flux through the IP_3_ receptor/channel and a constant leak flux. *c_1_* is the ratio between the volume of the ER and the volume of the cytosol. *c_ER_* and *c* are the Ca^2+^ in the ER and cytosolic Calcium, respectively; ν _1_ is the max Ca^2+^ channel flux, ν _2_ is the Ca^2+^ leak flux constant; ν _3_ is the Max Ca^2+^ uptake and *K_3_* is the Activation constant for ATP-Ca^2+^ pump (Table 2).

**Table 2:** Parameters of the De Young-Keizer model (Young et al., 1992).

Parameters of De Young-Keizer model		
Parameter	Value	Description
c_0_	2.0 μM	Total [Ca^2+^] in terms of cytosolic vol
c_1_	0.185	(ER vol)/(cytosolic vol)
ν_1_	6.0 s^−1^	Max Ca^2+^ channel flux
ν_2_	0.11 s^−1^	Ca^2+^ leak flux constant
ν_3_	0.9 μMs^−1^	Max Ca^2+^ uptake
k_3_	0.1 μM	Activation constant for ATP-Ca^2+^ pump
d_1_	0.13 μM	IP_3_
d_2_	1.049 μM	Ca^2+^ (inhibition)
d_3_	0.9434 μM	IP_3_
d_5_	0.08234 μM	Ca^2+^ (activation)
a_2_	0.2 μMs^−1^	Ca^2+^ (inhibition)
IP_3_	0.5 μM	IP_3_ flux

#### The Atri model

**Figure 5:**
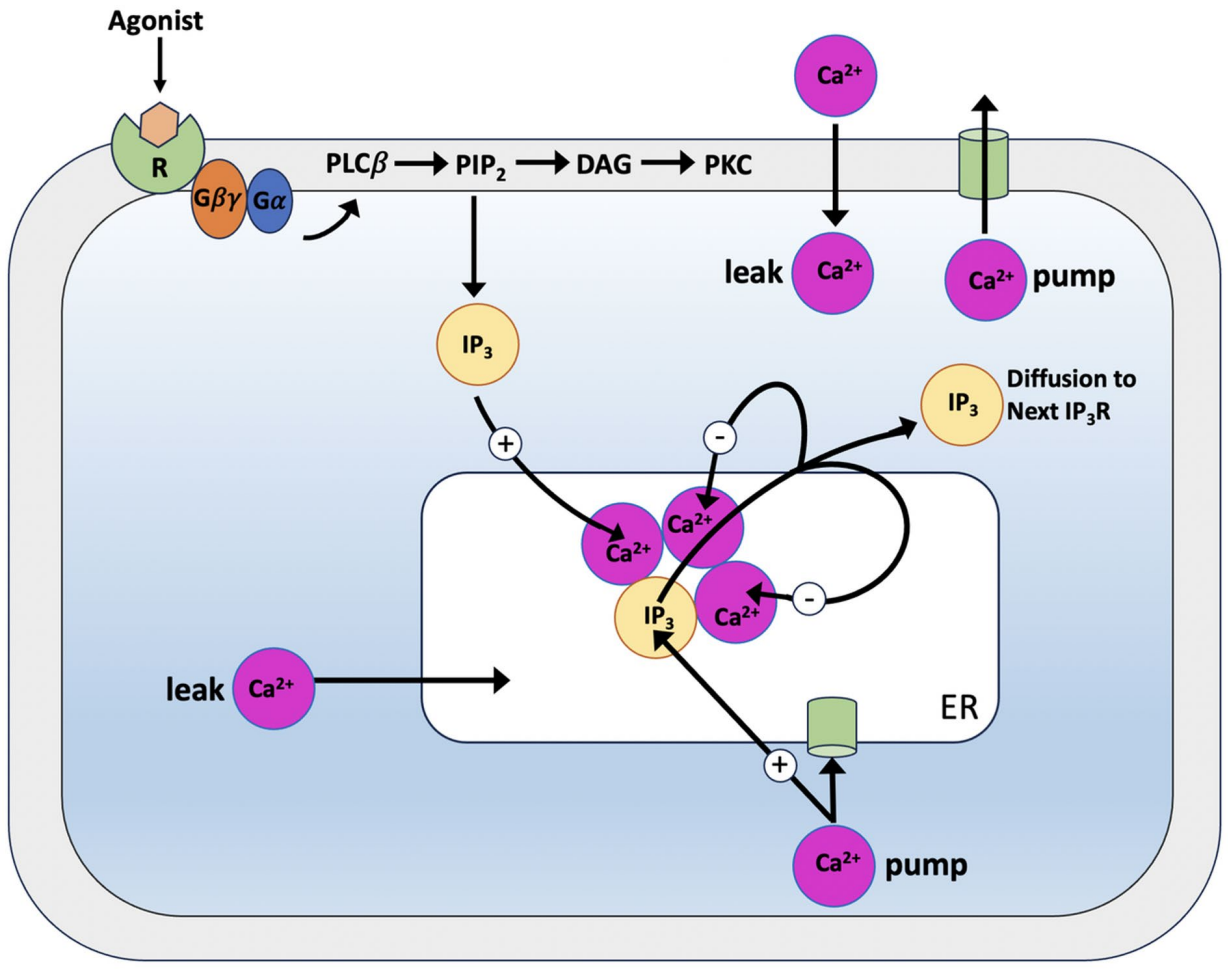
Schematic illustration of the Atri model. When IP_3_ reaches the binding sites of the IP_3_ receptor (IP_3_R), it allows Calcium to leave the endoplasmic reticulum by opening a Calcium-permeable channel. After leaving the channel, Calcium diffuses to the next storage site, inactivating the channel (−) and increasing (+) the sensitivity of the IP_3_R to IP_3_. Ca^2+^ pumps are used to return Calcium to the storage site.

In 1993, Atri et al. constructed a minimalist model for Ca^2+^ wave oscillations (Smrcka et al., 1991). The model, which served as the basis for a number of other models, proved simple enough to allow an understanding of the oscillatory phenomena underlying the spatio-temporal properties of Ca^2+^. A single intracellular Ca^2+^ pool that releases Ca^2+^ through the IP_3_R is included in the model. It is believed that Ca^2+^ modulates the IP_3_R in a biphasic manner, with intermediate Ca^2+^ acting to increase Ca^2+^ release while low and high Ca^2+^ act to block it (see Figure 5). The model takes its cue from Finch et al. (1991), and distinguishes between the time scales of channel activation and inactivation, where inactivation proceeds at a slower rate than activation. This temporal separation is critical for the spatial propagation of the Ca^2+^ signal, as inactivation must occur more gradually than activation to ensure the effective transmission of waves.

The model equation is:


(8) $$\frac{\mathrm{dc}}{dt}=J_{1}+J_{2}-J_{3}$$

According to Atri et al., there are three binding domains on the IP_3_ receptor, the first of which binds IP_3_ and the other two bind Ca^2+^; when IP_3_ is linked to domain 1 Ca^2+^ is attached to domain 2, but Ca^2+^ is not bound to domain 3, the receptor merely passes the Ca^2+^ current. Consequently, Ca^2+^ binds to domain 2 of the receptor to activate it and to domain 3 to deactivate it. Based on functionality, each binding domain consists of a certain number of binding sites. Assuming domain independence, the steady-state Ca^2+^ flux through the IP_3_ receptor, *J_1_*, is given by:


(9) $$J_{1}=k_{f}p_{1}p_{2}p_{3}$$

Where in Equation 9 $p_{1}$ is the probability that IP_3_ is bound to domain 1, $p_{2}$ is the probability that Ca^2+^ is bound to domain 2 and 1; $p_{3}$ is the probability that Ca^2+^ is bound to domain 3; $k_{f}$ is a constant and represents the maximum total Ca^2+^ influx through the IP_3_ receptors.

Thus, if we let *c* denote [Ca^2+^] can *P* denote [IP_3_] then the following Equations 9–19 result:


(10) $$p_{1}=\mu_{0}+\frac{\mu_{1}P}{k_{\mu }+P}$$

(11) $$p_{2}=b+\frac{V_{1}c}{k_{1}+c}$$

(12) $$p_{3}=1-\frac{c^{2}}{K_{2}^{2}+c^{2}}$$

Note that the expression of $p_{3}$ assumes that Ca^2+^ binds to the inactivating domain in a cooperative manner and while $p_{1}$ and $p_{2}$ are instantaneous functions of [Ca^2+^] and [IP_3_], $p_{3}$ acts on a slower time scale, therefore:


(13) $$J_{1}=k_{f}p_{1}p_{2}n$$

The dimensionless variable n represents the proportion of IP_3_ that have not been closed by Ca^2+^ and it is described by:


(14) $$\frac{dn}{dt}=\frac{n_{\infty }\left(c\right)-n}{\tau_{n}}$$

(15) $$n_{\infty }c=1-\frac{c^{2}}{k_{2}^{2}+c^{2}}$$

$n_{\infty }\left(c\right)$ is the steady-state value of n as a function of the intracellular Calcium concentration *c*,

$\tau_{n}$ is the time constant for the dynamics of n (Table 3).


(16) $$J_{1}=k_{\mathrm{flux}}\mu \left(\left[IP_{3}\right]\right)n\left(b+\frac{V_{1}c}{k_{1}+c}\right)$$

(17) $$J_{2}=\beta$$

(18) $$J_{3}=\frac{\gamma c}{k_{\gamma }+c}$$

(19) $$\mu \left(\left[IP_{3}\right]\right)=\mu_{0}+\frac{\mu_{1}\left[IP_{3}\right]}{k_{\mu }+\left[IP_{3}\right]}$$

**Table 3:** Parameters of the Atri model (Atri et al., 1993).

Parameters of Atri model		
Parameter	Value	Description
b	0.111	Proportion of IP_3_Rs spontaneously activated in the absence of bound Ca^2+^
V_1_	0.889	Proportion of IP_3_Rs that are activated by the binding of Ca^2+^
β	0.0–0.02 μMs^−1^	Constant rate of Ca^2+^ influx into the cytosol from the outside
γ	2.0 μMs^−1^	Maximum rate of Ca^2+^ pumping from the cytosol
τ_n_	2.0 s	Time constant for the dynamics of n, the proportion of IP_3_Rs not closed by Ca^2+^
k_1_	0.7 μM	Constant related to the activation of a channel in response to Calcium binding
k_γ_	0.1 μM	[Ca^2+^]c at which the rate of Ca^2+^ pumping from the cytosol is at half-maximum
k_2_	0.7 μM	Constant related to the inactivation of a channel in response to Calcium binding
k_flux_	8.1 μMs^−1^	Maximum total Ca^2+^ flux through all IP_3_Rs

#### The Li and Rinzel model

**Figure 6:**
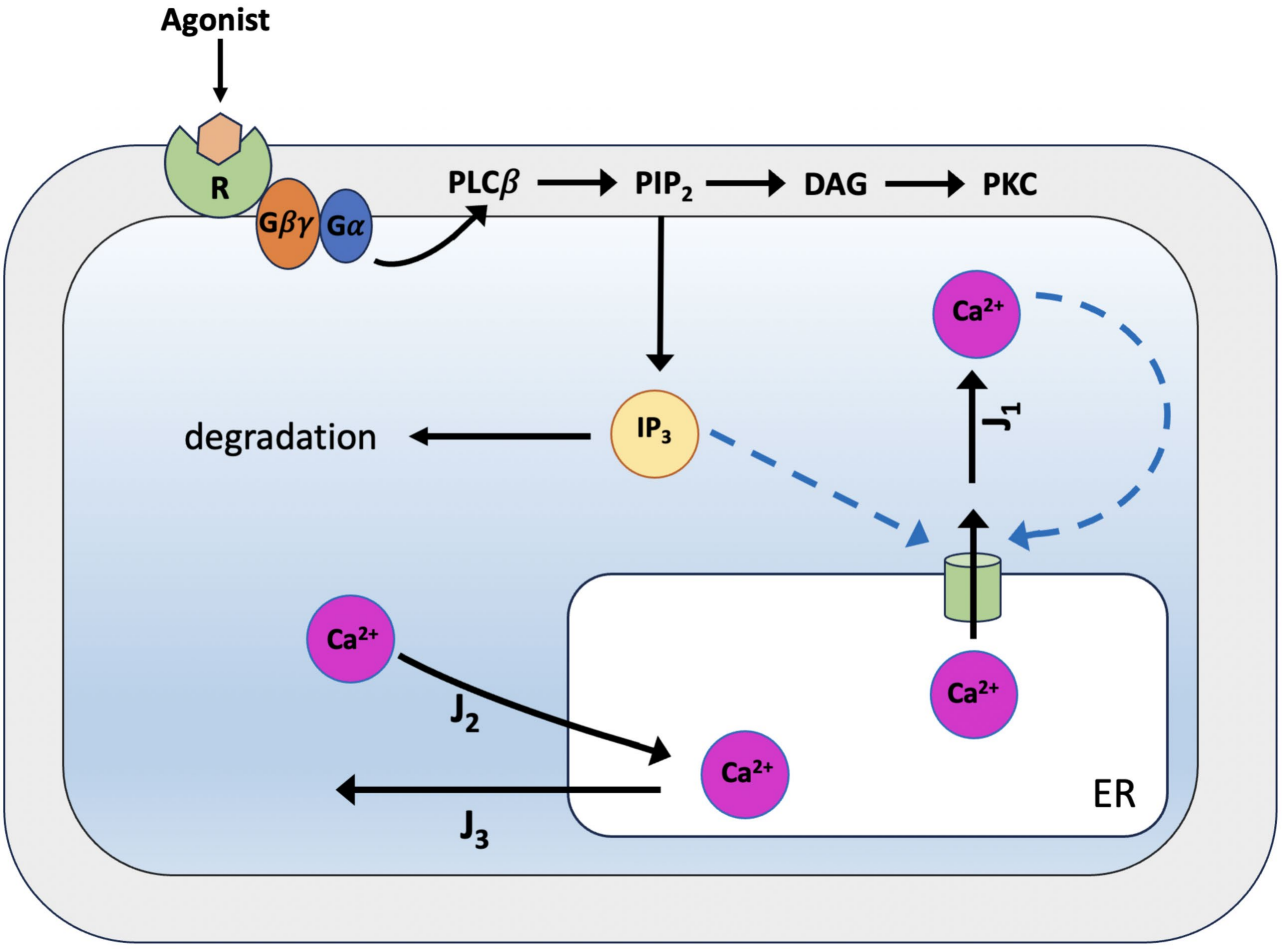
Schematic representation of Calcium dynamics according to the Li-Rinzel model. The model focuses on an individual cell situated within an extracellular environment devoid of Ca^2+^, thus negating the influx and efflux of Ca^2+^ through the cell membrane. Consequently, the intracellular Ca^2+^ dynamics are prompted by IP_3_, which is initially required to open the IP_3_ receptors on the ER membrane and prime the channels for Calcium-mediated feedback activation in the cytoplasm. Subsequently, the Calcium dynamics are governed by the interplay between Calcium-induced Calcium release (CICR), a non-linear amplification process regulated by the Calcium-dependent opening of channels to the ER’s Calcium stores, and the activity of the active SERCA pumps, which facilitate a reverse flow. Basal Ca^2+^ levels, on the other hand, are determined by the balance between a nonspecific passive loss of Ca^2+^ from the ER stores into the cytoplasm and the active uptake by SERCA pumps.

In 1994, Yue-Xian Li and John Rinzel deduced a model that reduces the De Young-Keizer model to a two-variable system to describe Calcium dynamics. This was mainly done by identifying the binding rates involving IP_3_ and activating Ca^2+^ molecules as faster rates than the binding rate of deactivating Ca^2+.^ This made it possible to essentially split the model into two halves, with and without deactivating Ca^2+^ binding. The two dynamic variables of the LR model are the concentration of free cytosolic Ca^2+^ (*C*) and the fraction of open subunits of the inositol triphosphate receptor (*h*) (see Figure 6; Li and Rinzel, 1994); this result was obtained by using the method of multiple scales to solve the equations of the De Young-Keizer model on a succession of faster time scales to reduce it to a 2D system:


(20a) $$\frac{\mathrm{dc}}{dt}=J_{1}+J_{2}-J_{3}$$

and


(21a) $$\frac{dh}{dt}=\frac{h_{\infty }-h}{\tau_{h}}$$

with J1, J2, and J3 given by the equations:


(22) $$J_{1}=c_{1}\nu_{1}m_{\infty }^{3}h^{3}\left(c_{ER}-c\right)$$

(23) $$J_{2}=c_{1}\nu_{2}\left(c_{ER}-c\right)$$

(24) $$J_{3}=\frac{\nu_{3}c^{2}}{c^{2}+k_{3}^{2}}$$

Were *J_1_* is a release of Ca^2+^, mutually controlled by Ca^2+^ and by IP_3_ concentration; *J_2_* is a passive loss of Ca^2+^ from the endoplasmic reticulum (ER) to the cytosol; and *J_3_* an active absorption of Ca^2+^ in ER due to the action of the pumps. Again, in Equation 22, $h=x_{000}+x_{100}+x_{010}+x_{110}$ is the fraction of channel not yet inactivated by Ca^2+^.

Along with the gating variables:


(25) $$m_{\infty }=\frac{I}{I+d_{1}}\;\frac{C}{C+d_{5}}$$

(26) $$h_{\infty }=\frac{Q_{2}}{Q_{2}+C}$$

(27) $$T_{h}=\frac{1}{a_{2}\left(Q_{2}+C\right)\;}$$

(28) $$Q_{2}=\frac{I+d_{1}}{I+d_{3}}d_{2}$$

Therefore, the level of IP_3_ is directly controlled by the signals affecting the cell from its external environment. In turn, the level of IP_3_ determines the dynamic behavior of the LR model. The Calcium signal can therefore be considered as coded information relating to the level of IP_3_ (Table 4).

**Table 4:** Parameters of the Li-Rinzel model (Li and Rinzel, 1994).

Parameters of Li-Rinzel model		
Parameter	Value	Description
c_0_	2.0 μM	Total [Ca^2+^] in terms of cytosolic vol
c_1_	0.185	(ER vol)/(cytosolic vol)
ν_1_	6.0 s^−1^	Max Ca^2+^ channel flux
ν_2_	0.11 s^−1^	Ca^2+^ leak flux constant
ν_3_	0.9 μMs^−1^	Max Ca^2+^ uptake
k_3_	0.1 μM	Activation constant for ATP-Ca^2+^ pump
d_1_	0.13 μM	IP_3_
d_2_	1.049 μM	Ca^2+^ (inhibition)
d_3_	0.9434 μM	IP_3_
d_5_	0.08234 μM	Ca^2+^ (activation)
a_2_	0.2 μMs^−1^	Ca^2+^ (inhibition)
IP_3_	0.5 μM	IP_3_ flux

Most models for Ca^2+^ dynamics are derived from the two-variable models mentioned so far. Since the realization of the pioneering models mentioned above, the intracellular dynamics of Ca^2+^ and IP_3_ have been characterized much more comprehensively, and above all, specific and more sophisticated models for intracellular and extracellular Ca^2+^ dynamics in astrocytes have been realized. When astrocytes respond to stimulation, they register a variety of spatiotemporal dynamics of Ca^2+^ elevation, each of which may have its own coding. Understanding the biophysical mechanisms underlying the rich Ca^2+^ dynamics in astrocytes is important because distinct coding patterns may correspond to different downstream signaling, including gliotransmission and consequently control of synaptic function.

More recently, models have also been created for subcellular Ca^2+^ increases linked to metabotropic glutamate receptors (mluRs). Here, the models offer the possibility of establishing a link between the properties of mGluRs and their implication in intracellular Ca^2+^ dynamics.

Glutamate is the most abundant excitatory neurotransmitter in the brain and plays a crucial role in various physiological processes, including learning, memory, and synaptic plasticity.

As demonstrated by electron microscopy the outer surface of the plasma membrane of astrocytes has specific receptors for glutamate. Smith et al. (2014) showed that cultured astrocytes responded to extracellular glutamate with rapid and oscillatory elevations of intracellular free Ca^2+^ concentration (Innocenti et al., 2000; Dani et al., 1992; Charles et al., 1991). In 1994, Mennerick and Zorumski experimentally demonstrated that astrocytes are able to uptake and transport 90% of glutamate from the extracellular space (Mennerick and Zorumski, 1994); Parpura and Haydon subsequently demonstrated that astrocytes modulate neuronal excitability through the release of glutamate linked to physiologically relevant increases in Ca^2+^ (Shao and Mccarthy, 1994; Parpura and Haydon, 2000).

Metabotropic glutamate receptors (mGluRs) are membrane proteins capable of responding to glutamate, the central nervous system’s main excitatory neurotransmitter as a result, they are crucial in the transmission of signals between cells in the nervous system. Research employing *in situ* hybridization and immunocytochemistry reveals that mGluR3 is the most often expressed mGluR subtype in glia (Smith, 1992; Petralia et al., 1996; Wroblewska et al., 1998). Astrocytes express Group I mGluR subtypes, which includes mGluR1 and 5, reviews can be found in Barres, 1991 (Barres, 1991; Steinhäuser and Gallo, 1996; Parpura et al., 1994; Testa et al., 1995; Miller et al., 1995; Winder and Conn, 1996; Hermans and Challiss, 2001; Verkhratsky et al., 1998; Biber et al., 1999; Cai et al., 2000; Aronica et al., 2003; Perea and Araque, 2007; Araque and Navarrete, 2010; Sun et al., 2013).

It is interesting to note that cell lines that express the mGluR5 receptor are the primary source of concurrent InsP3 and Ca^2+^ oscillations. These glutamate-induced Ca^2+^ oscillations have unusual characteristics, so it is plausible that different oscillatory mechanisms prevail depending on the receptor type (Kummer et al., 2000; Lemon et al., 2003; De Pittà et al., 2009).

When glutamate binds to its membrane receptor, a sequence of events is set off: the ethorotrimeric G-protein, which is named for its three distinct polypeptide subunits, *α*, *β*, and *γ*, interacts with the receptor to create a receptor-G-protein complex on the inner membrane surface. When the *α* subunit interacts with the receptor, it undergoes a conformational shift that releases the GDP attached to it and replaces it with GTP. This, in turn, activates the phospholipase C-β (PI-PLCβ) that is specific to phosphatidyl-inositol. PI-PLCβ is located on the inner surface of the membrane, linked to the interaction between its PH domain and a PIP_2_ molecule immersed in the bilayer. The PI-PLCβ enzyme catalyzes a reaction that cleaves PIP_2_ into two molecules, inositol 1,4,5-triphosphate (IP_3_) and diglycerol (DAG). The resultant IP_3_ molecules diffuse into the cytoplasm and attach to a particular IP_3_ receptor found on the smooth endoplasmic reticulum surface (Rosa et al., 2022). DAG stimulates PKC activity, which in turn phosphorylates the mGlu5 receptor at Ser-839. This phosphorylation leads to the uncoupling of the receptor from the G protein signaling cascade.

Modeling studies have not always been conducted in tandem with experimental research on mGlur receptor-mediated Ca^2+^ signaling; although mGlur receptors are highly expressed in the central nervous system (CNS) and have been linked to several pathophysiological processes as well as neuro-psychiatric disorders (Nicoletti et al., 2011; Spooren et al., 2001).

#### The De Pittà model

**Figure 7:**
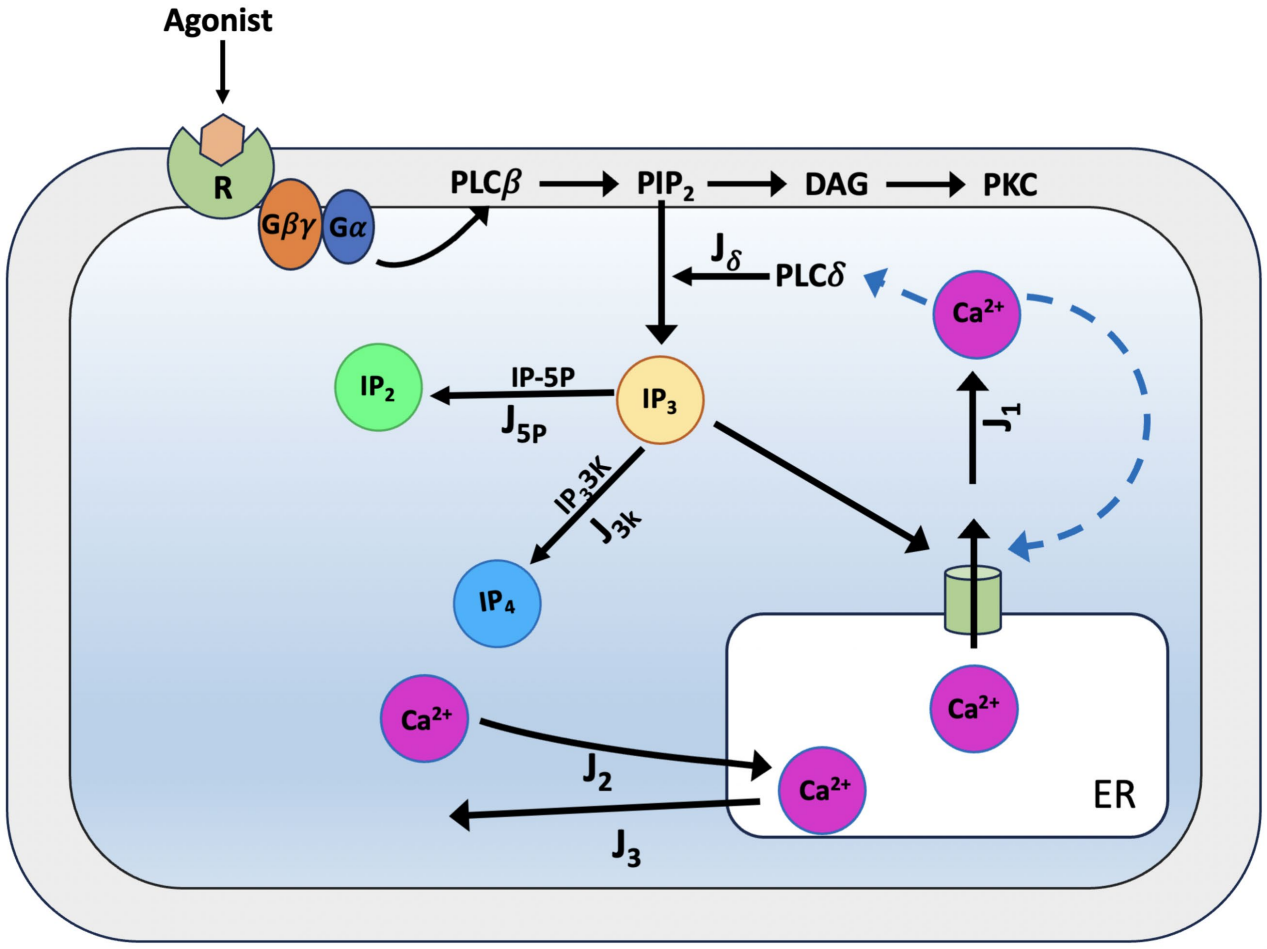
Schematic representation of Ca^2+^ dynamics and IP_3_ production according to the De Pittà model. When glutamate binds to metabotropic glutamate receptors (mGluR1/5), the receptor activates a G_q_ protein, which subsequently stimulates phospholipase C-beta (PLC-β). PLC-β hydrolyses phosphatidylinositol 4,5-bisphosphate (PIP_2_) into two second messengers: inositol 1,4,5-trisphosphate (IP_3_) and diacylglycerol (DAG). IP_3_ diffuses into the cytoplasm and binds to endoplasmic reticulum (ER) receptors (*J_δ_*), triggering the release of Calcium ions (Ca^2+^) and initiating downstream cellular responses. IP_3_ can then be degraded by IP_3_-3-kinase (IP_3_-3 K) (*J_3K_*) to inositol 1,3,4,5-tetrakisphosphate (IP_4_) or by inositol polyphosphate 5-phosphatase (IP-5P) (*J_5p_*) to inositol 1,4-bisphosphate (IP_2_), modulating the signaling cascade. For the CICR, the model considers the original formulation of the Li-Rinzel model (Li and Rinzel, 1994).

Young et al. (1992), Li and Rinzel (1994), and Höfer et al. (2002) models as a starting point, in De Pittà et al. (2009) constructed a generic model for glutamate-induced Ca^2+^ (Glu) dynamics in astrocytes, including additional biochemical processes relevant for a more realistic description of astrocyte activity. Such extensions include the production and degradation of IP_3_ within the astrocyte cell, mediated by two membrane-associated enzymes, PLCβ and PLCδ (see Figure 7). Later, De Pittà and Berry (2019) further refined their model by focusing on the rate of IP_3_ production following activation of glutamate receptors mGluRs, building a new model. The De Pittà model for IP_3_/Ca^2+^ signaling is constituted by three ODES, respectively, for intracellular Ca^2+^ (C), the IP_3_R gating (h), and the mass balance equation for intracellular IP_3_ lumping terms. Regarding the differential equations for the variables *C* and *h* above, the De Pittà model considers the original Li-Rinzel model formulation described for the CICR and provides a more detailed description of IP_3_ production and degradation, proposing a three-variable model for glutamate-induced intracellular Calcium dynamics caused by synaptic activity in astrocytes.


(20b) $$\frac{\mathrm{dc}}{dt}=J_{1}+J_{2}-J_{3}$$

(21b) $$\frac{dh}{dt}=\frac{h_{\infty }-h}{\tau_{h}}$$

In astrocytes, IP_3_ together with diacylglycerol (DAG) is produced by hydrolysis of phosphatidylinositol 4,5-bisphosphate (PIP_2_) by two phosphoinositide-specific phospholipase C (PLC) isoenzymes, PLCβ and PLCδ (Rebecchi and Pentyala, 2000). PLCβ is primarily controlled by cell surface receptors; hence, its activity is linked to the level of external stimulation (i.e., the extracellular glutamate) and as such, it pertains to the glutamate-dependent IP_3_ metabolism. PLCδ is the enzyme responsible of endogenous IP_3_ production in astrocytes, it is essentially activated by increased intracellular Ca^2+^ levels (Rhee and Bae, 1997). The model proposed for PLCδ -mediated IP_3_ production (*J_δ_*) (Equation 29) derived from structural and mutational studies (Höfer et al., 2002; Pawelczyk and Matecki, 1998).


(29) $$J_{\delta }=O_{\delta }\left(1-\frac{I}{I+K_{\delta }}\right)\left(\frac{c^{2}}{c^{2}+K_{\delta }}\right)$$

where *O_δ_* is the maximal rate of IP_3_ production by PLCδ and *K_δ_* is the inhibition constant of PLCδ activity. According to experiments, PLCδ activity is inhibited by high IP_3_ concentrations (> 1 μM) because they compete with PIP_2_ for the enzyme’s binding (Allen and Barres, 2009).

In astrocytes there are two several pathways for IP_3_ degradation: the dephosphorylation of IP_3_ by inositol polyphosphate 5-phosphatase (IP-5P), and the phosphorylation of phosphorylation of IP_3_ by the IP_3_3-kinase (IP_3_-3 K). For the description of the two IP_3_ degradation dynamics we use the relations given by Equations 30, 31:


(30) $$J_{5p}=O_{5p}\left(\frac{I}{I+K_{5p}}\right)$$

where *O_5p_* is the maximal rate of IP-5P mediated IP_3_ degradation in the linear approximation.

For IP_3_-3 K degradation we can write:


(31) $$J_{3K}=O_{3K}\left(\frac{c_{4}}{c_{4}+K_{3K}^{4}}\right)\left(\frac{I}{I+K_{3K}}\right)$$

where *O_3k_* is the maximal rate of IP_3_ degradation by IP_3_-3 K.

In summary, the De Pittà model of Ca^2+^ dynamics with endogenous IP_3_ metabolism (Equation 32) is based on the two LR equations but the IP_3_ concentration (I) is now provided by a third coupled differential Equations 20a, 20, 21, 21b, 33.


(32) $$\frac{dI}{dt}=J_{\delta }-J_{5p}-J_{3K}$$

(33) $$\begin{array}{l} \frac{dI}{dt}=O_{\delta }\left(1-\frac{I}{I+K_{\delta }}\right)\left(\frac{c^{2}}{c^{2}+K_{\delta }}\right)-O_{5p}\left(\frac{I}{I+K_{5p}}\right)-\\ O_{3K}\left(\frac{c_{4}}{c_{4}+K_{3K}^{4}}\right)\left(\frac{I}{I+K_{3K}}\right) \end{array}$$

The model highlights the complex biochemical reactions coupled with Ca^2+^ dynamics via the different second messengers (Table 5).

**Table 5:** Parameters of the De Pittà model (De Pittà and Berry, 2019).

Parameters of De Pittà model		
Parameter	Value	Description
c_0_	10.0 μM	Total [Ca^2+^] in terms of cytosolic vol
ν_1_	7.759 s^−1^	Maximal Ca^2+^ release rate by IP_3_Rs
ν_2_	0.01 s^−1^	Ca^2+^ leak rate
O_2_	0.325 μM^−1^ s^−1^	Ca^2+^ leak rate
k_3_	0.1 μM	Ca^2+^ affinity of SERCA pumps
ν_3_	10.0 μMs^−1^	Maximal Ca^2+^ uptake rate
d_1_	0.1 μM	IP_3_
d_2_	4.5 μM	Ca^2+^ (inhibition)
d_3_	0.1 μM	IP_3_
d_5_	0.05 μM	Ca^2+^ (activation)
c_1_	0.5	ER-to-cytoplasm volume ratio
O_β_	0.141 μMs^−1^	Maximal rate of IP_3_ production by PLCβ
Γ_A_	1.0	Fraction of bound receptors
O_δ_	0.05 μMs^−1^	Maximal rate of IP_3_ production by PLCδ
K_δ_	0.5 μM	Ca^2+^ affinity of PLCδ
k_δ_	1.0 μM	Inhibiting IP_3_ affinity of PLCδ
Ω_5P_	0.86 s^−1^	Rate of IP_3_ degradation by IP-5P
O_3K_	0.163 μMs^−1^	Maximal rate of IP_3_ degradation by IP_3_3K
K_3K_	1.0 μM	IP_3_ affinity of IP_3_3K
K_D_	0.5 μM	Ca^2+^ affinity of IP_3_3K

## Data sources

**Figure 8:**
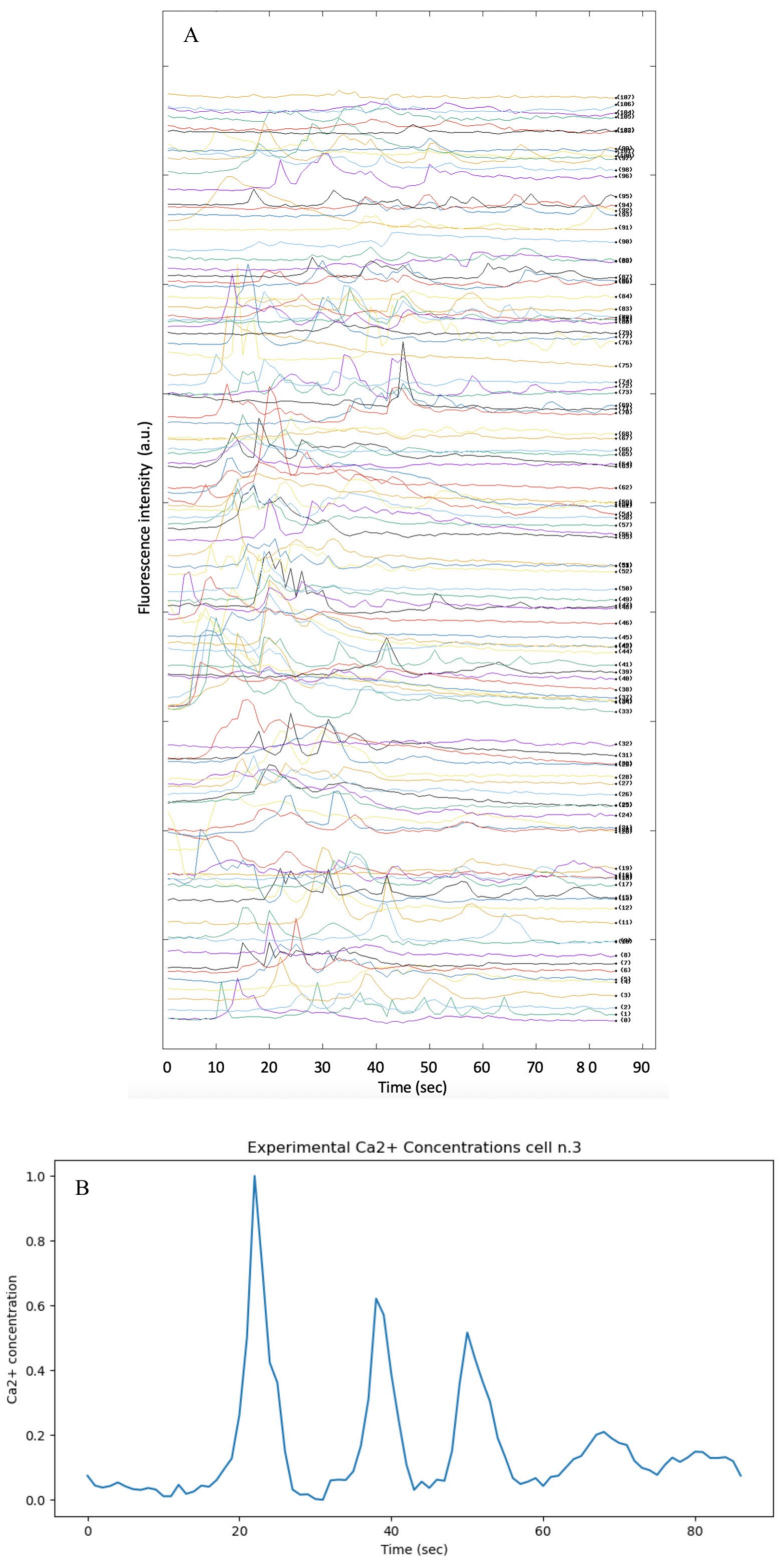
Fluorescence intensity profiles showing the spontaneous activity of intracellular Ca^2+^ in immortalized human astrocytes. The methodology and instrument described in Musotto et al. (2024) was used to produce the experimental data. **(A)** Split image of each Ca^2+^ cell present in the well under analysis. The Ca^2+^ signal of each cell present in the well was split in order to provide all data simultaneously. **(B)** Intensity vs. time profile of Ca^2+^ fluorescence intensity in cell n.3.

For the acquisition of experimental data, the methodology described in the article “Dynamics of Astrocytes Ca^2+^ Signaling: A Low-Cost Fluorescence Customized System for 2D Cultures” was adopted (Musotto et al., 2024), this study provides temporal and spatial data of Calcium signaling in astrocytes using an innovative and inexpensive fluorescence imaging system designed for two-dimensional (2D) cell cultures. The analysis was performed on immortalized human astrocytes, the raw data for all cells in the well analyzed are shown in Figure 8.

The background was subtracted from the raw data and normalized by calculating the change in fluorescence (ΔF) from baseline fluorescence (F₀) (Wamhoff et al., 2002). This normalization process is essential to ensure that the data reflect true physiological changes rather than artifacts introduced by variable dye loading.

In order to visualize the variables on different scales and to facilitate comparison between theoretical and experimental data, all data were scaled by the min-max normalization method in the range [0,1]. In order to compare the theoretical Ca^2+^ signal obtained from the models reported in the article, cell no. Three was chosen arbitrarily (see Figure 8B)

Comparing model predictions with experimental data makes it possible to assess the accuracy and reliability of models, identify discrepancies and refine models accordingly.

## Results

### The Goldbeter model

**Figure 9:**
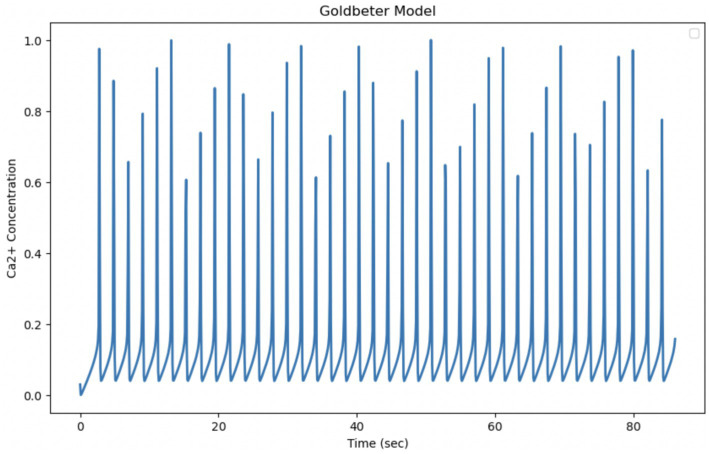
Simulation of the Golbeter model (Goldbeter et al., 1990). Curves obtained by integrating Equations 1 and 2 with the parameters shown in Table 1. Fluctuations of cytosolic Ca^2+^ concentration in 87 s, a time comparable to the experimental observation time. Goldbeter et al. obtained the reported fluctuations with an external stimulation *β* of 30.1%.

The Goldbeter model is known to describe intracellular Calcium oscillations, which in many biological situations exhibit regular and periodic behavior, but is highly sensitive to the parameters that govern it; in this form, it appears to be insufficient to explain the experimental data on Ca^2+^ dynamics in astrocytes. The theoretical model, as reported in the original article, describes the Calcium dynamics over a shorter time interval (10 s), while the experimental data cover a longer period (87 s). By extending the integration time of the model to 87 s, so as to be comparable with the experimental time, it can be observed that the Z oscillations persist throughout the interval with a fairly stable amplitude and frequency. The oscillations do not disappear and the system does not converge to a static equilibrium, but seems to maintain a repetitive oscillation pattern. The pattern is set to produce sustained oscillations that continue for longer times. The parameters of the pattern determine how fast Calcium enters, is released and is removed from the various compartments of the cell. To adapt the model to the much slower experimental Ca^2+^ dynamics, the model parameters must be modified. The experimental data provided show less regular behavior and more unpredictable amplitude variations. The large differences observed suggest that the actual biological system is more complex and requires optimization of model parameters or more refined modeling (Figure 9).

### The Atri model

**Figure 10:**
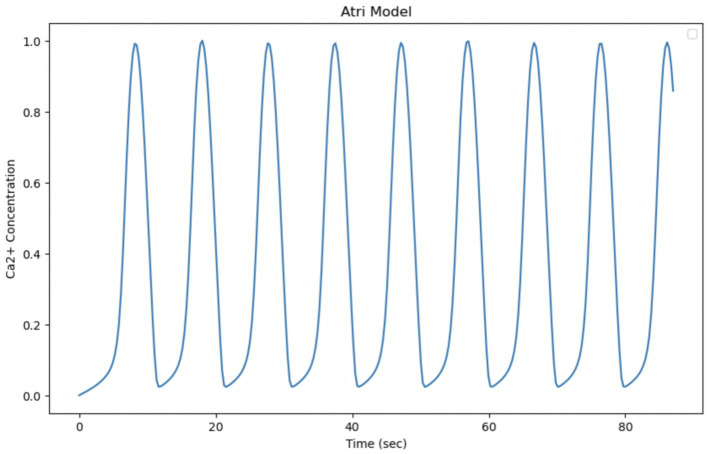
Simulation of the Atri model. Curves obtained by integrating Equations 8, 19 with the parameters shown in Table 3. Oscillation of cytosolic Ca^2+^ concentration in 87 s, time comparable with experimental observation time.

The Atri model is based on a simplified system of differential equations that mainly considers the release and pumping of intracellular Calcium. By extending the simulation of the model to make it temporally comparable with experimental data, whose observation time is equal to 87 s, it can be seen that the oscillations are regular, with stable amplitude and average frequency. The experimental data, on the other hand, show changes in the behavior of Calcium over a period of 87 s, with an initial activation phase, a maximum peak, and a subsequent decline. This indicates that the biological system may have richer temporal dynamics that the model cannot fully reproduce. These discrepancies suggest that the model, in its current form, fails to fully capture the complexity of the experimental behavior of intracellular Calcium in astrocytes. A key factor in the Atri model is the gating variable *n*, which regulates the opening of Calcium release channels. This variable introduces a feedback mechanism that can influence the frequency of oscillations, making the model more flexible with respect to the timing of oscillation (Figure 10).

### The Li-Rinzel model

**Figure 11:**
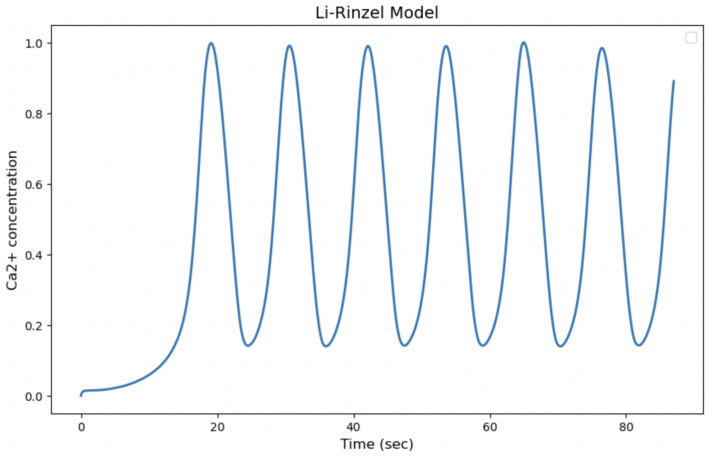
Simulation of the Li-Rinzel model. Curves obtained by integrating Equations 20, 21 with the parameters shown in Table 4. Oscillation of cytosolic Ca^2+^ concentration in 87 s, time comparable with experimental observation time.

The Li-Rinzel model originates from a reduction of the more complex model of De Young and Keizer, with the aim of simplifying the description of intracellular Calcium oscillations while maintaining the ability to reproduce experimentally observed phenomena. The model is particularly useful for describing the regulation of Calcium release via IP₃ receptors in the endoplasmic reticulum. It explicitly introduces the Calcium concentration in the endoplasmic reticulum as a dynamic variable, which makes it more detailed in its description of the Calcium release and reabsorption cycle and capable of reproducing more regular and structured oscillations than simpler models. The ability of the model to generate slow Ca^2+^ input-dependent oscillations, as in Figure 5 of the article “Equations for InsP, Receptor-mediated [Ca^2+^], Oscillations Derived from a Detailed Kinetic Model: A Hodgkin-Huxley Like Formalism,” makes it more suitable for comparison with our experimental data on Ca^2+^ signaling in astrocytes. However, the regularity of oscillations predicted by the model may be less realistic than experimentally observed oscillations, which tend to be more irregular and less predictable (Figure 11).

### The De Pittà model

**Figure 12:**
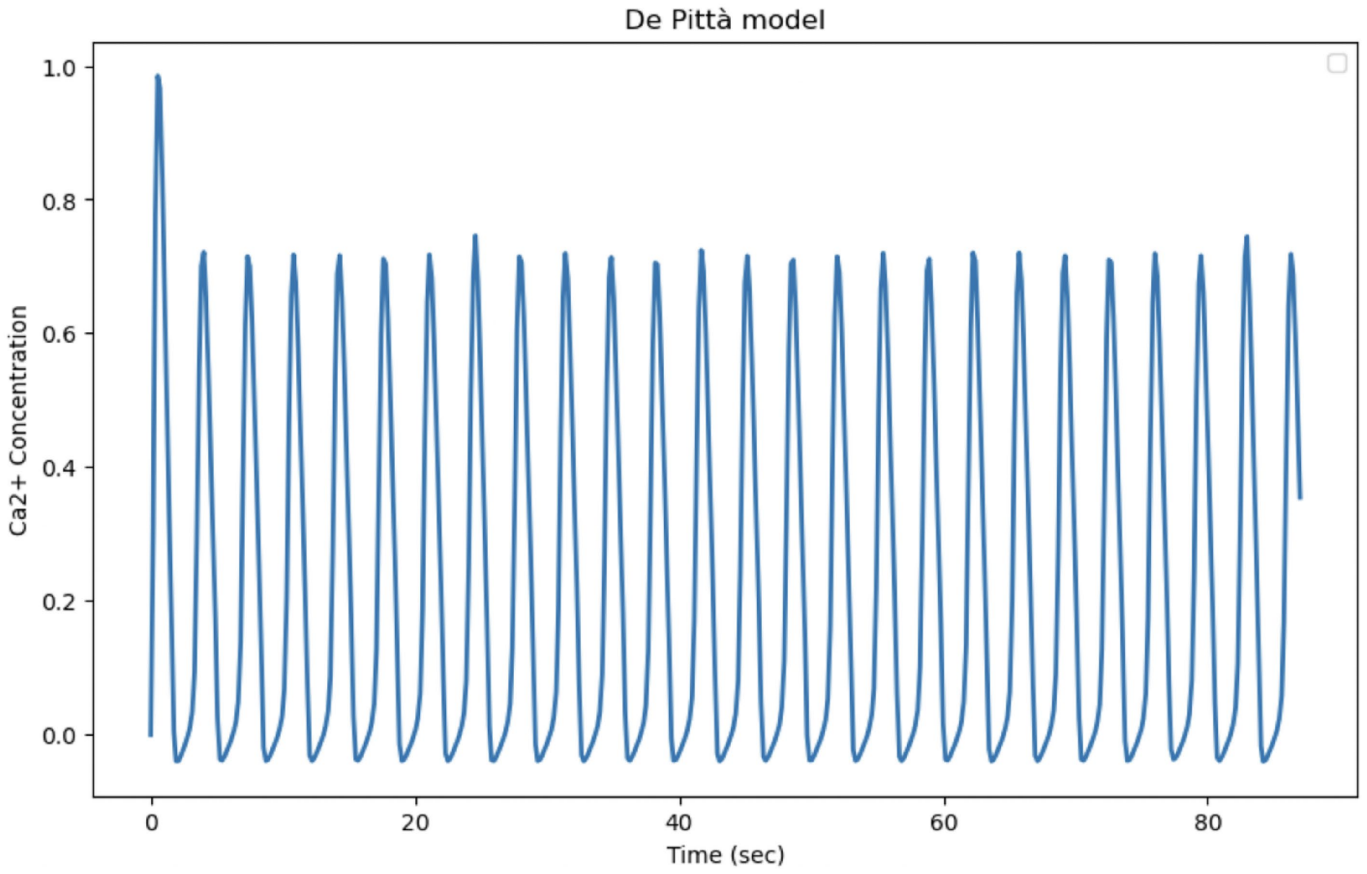
Reproduction of the De Pittà model. Curves obtained by integrating Equations 20, 21 of the Li-Rinzel model relating to Calcium-induced Calcium dynamics (CIRC) with Equation 31 relating to IP3 production and degradation. The model parameters are shown in Table 4. Oscillation of cytosolic Ca^2+^ concentration of the model in 87 s, time comparable with experimental observation time.

The De Pittà model is a powerful tool to describe intracellular Calcium oscillations regulated by G-protein-coupled receptors. In the model, G-protein-coupled receptors, when activated, induce the release of IP₃, which in turn stimulates the release of Calcium from the endoplasmic reticulum. The released Calcium can further activate Calcium release channels through the process of induced Calcium release (CICR), creating positive feedback. Like many other Calcium oscillation models, De Pittà includes positive feedback (via CICR) and negative feedback (via Calcium reabsorption in the endoplasmic reticulum or degradation of the IP₃ signal). These mechanisms are crucial for the generation of regular oscillations. Although it provides a realistic description of IP_3_ and CICR mediated Calcium release, it has some limitations compared to experimental data, particularly with regard to its ability to capture the irregularity and variability of Calcium oscillations. The experimental data show much more dynamic and complex behavior, with significant variations in amplitude and frequency that the model does not fully reproduce in its current form. In order to have a better fit to the experimental data, the parameters could be calibrated. Optimization of Calcium release and absorption rates, as well as IP₃ dynamics, could improve the fit of the model (Figure 12; Table 6).

**Table 6:** This table summarizes the main features, advantages, and limitations of the Goldbeter, De Young-Keizer, Atri, Li-Rinzel, and De Pittà models, providing with a quick reference to understand the merits and constraints of each modeling approach.

Comparative overview of mathematical model for Calcium dynamics			
Model	Main Features	Advantages	Limitations
Goldbeter	A minimalistic model for calcium oscillations based on enzymatic feedback	Simple and intuitive; highlights basic oscillatory mechanisms.	Does not capture specific details of IP_3_ receptors and more complex molecular interactions.
De Young-Keizer	Provides a detailed description of the IP3 receptor with multiple states (activation and inhibition) and calcium dynamics.	Offers a realistic and in-depth representation of the IP_3_/Ca^2+^ system.	Highly complex with many parameters, making analysis and calibration challenging.
Atri	A simplified model that integrates both positive and negative feedback in the IP3-Ca^2+^ system.	Facilitates theoretical analysis and bifurcation studies thanks to its reduced structure.	The simplification may overlook some relevant molecular details.
Li-Rinzel	A reduced version of the De Young-Keizer model that retains the essential dynamics of calcium oscillations.	Balances key mechanism simplicity with ease of mathematical analysis	Balances key mechanism simplicity with ease of mathematical analysis
De Pittà	Integrates molecular and spatial aspects, making it particularly suitable for simulating complex dynamics (e.g., in astrocytes).	Provides a comprehensive and versatile approach to simulate complex interactions in physiological contexts.	High computational complexity and numerous parameters make calibration challenging.

## Discussion

Over the past 20 years, many computational models for intracellular Ca^2+^ dynamics have been developed. They differ according to the level of description, from the microscopic level, for which stochastic models must be used, to the macroscopic level, which requires deterministic models. In this review, five models of intracellular Ca^2+^ dynamics were evaluated (Goldbeter et al., 1990; Young et al., 1992; Atri et al., 1993; Li and Rinzel, 1994; De Pittà and Berry, 2019), implementing the equations based on what was presented in the original publications. Our aim was to reproduce the simulation results of the original articles and compare them with the experimental data in our possession (Musotto et al., 2024) in order to determine which model was most suitable.

The aim of the mathematical models analyzed in this contribution is to interpret the emergence of complex intracellular Calcium dynamics as the result of interdependent Ca^2+^ fluxes between the cytosol and intracellular stores, driven by the interaction with IP_3_. The models are described by systems of non-linear ordinary differential equations (ODEs), which are capable of supporting self-sustained Calcium oscillations. These phenomenological models have been developed to reproduce Calcium flow behavior comparable with available experimental data and have played a crucial role in the advancement of neuroscience, serving as a bridge between experimental observations and the development of more in-depth theories. All models discussed here are described by deterministic equations, meaning that the effects of stochastic fluctuations due to microscopic inhomogeneities and noise due to spatial localization or random fluctuations are neglected. Indeed, one of the limitations of deterministic models is that they produce oscillations that are too regular compared to those observed experimentally. The addition of stochastic components could improve the models’ ability to fit the experimental data. It has been shown that IP_3_ channels are distributed in clusters on the ER membrane, generating Ca^2+^ signals on multiple scales, ranging from local puffs to global intra- and extracellular waves. It should be pointed out that our observation of intracellular Ca^2+^ dynamics in astrocytes is given by whole-cell oscillations. These signals are believed to include release from the multiple compartmentalized processes within the cell (Bindocci et al., 2017; Smith and Parker, 2009) that give rise to the observed global Ca^2+^ oscillations.

From the study of the models reported in this paper, a common problem emerges: the period of intracellular Ca^2+^ fluctuations are faster than that observed experimentally. The period of Calcium fluctuations in astrocytes is generally slower, often occurring within seconds or minutes. Research indicates that Calcium signals in astrocytes can be attributed to delayed release from internal stores, leading to slower kinetics than in neuron (Ma et al., 2021). Furthermore, astrocyte Calcium transients can be influenced by various signaling pathways, including those mediated by inositol trisphosphate (IP_3_) and ryanodine receptors, which contribute to the complexity and variability of astrocyte Calcium dynamics (Stobart et al., 2018; Corkrum et al., 2019). The frequency and duration of these Calcium events can vary significantly depending on the physiological state of the astrocytes and the surrounding neuronal activity (Schnell et al., 2011; Mcdougal et al., 2013). In summary, while Calcium fluctuations in some cells and neurons are rapid and occur on the millisecond scale, astrocyte Calcium signaling operates on a slower time scale, typically between seconds and minutes, with the possibility of intercellular propagation of Calcium waves. Of the five models studied, only four were implemented, as the Li-Rinzel model is a simplification of the De Young-Keizer model and it was decided not to simulate the latter because it was too computationally expensive. The models analyzed in this review were simulated using the parameters reported in the original studies. Each model has unique mechanisms and parameters that influence the dynamics of Ca^2+^ signaling. A general comparison of the four implemented models shows that they have different abilities to modulate Ca^2+^ frequencies, varying in complexity and adaptability. Goldbeater model generates constant frequencies that depend on the parameters of Ca^2+^ release and accumulation without direct influence from IP_3_. This model is suitable for constant and rhythmic cellular responses. In Atri model, the frequency of oscillations varies depending on the levels of IP_3_ and the rate of binding of IP_3_ to its receptors. The oscillations are influenced by spatial diffusion, which allows variable frequencies and the formation of Ca^2+^ waves ideal for complex communications between different cellular regions. In Li and Rinzel model, the frequency of Ca^2+^ oscillations are dependent on the concentration of IP_3_. As IP_3_ increases, the frequency of Ca^2+^ oscillations also increase. This model is used to analyze cellular responses that must vary gradually with external stimuli. The most complex of the models analyzed, the G-ChI model, describes the Ca^2+^ frequency as a result of the dynamic interaction between Ca^2+^, IP_3_, and GPCR receptors. Oscillations in this model respond to different synaptic stimuli, with frequencies modulated by enzymes such as PLC and PKC. It therefore allows a highly adjustable frequency, ideal for neurobiological functions in astrocytes.

In the present contribution, a comparison of five models representing different modeling approaches, with the aim of identifying which of these bests fit our experimental data. The selected model will be used as a basis for developing a model with a structure derived from the application of physical principles as in Gawthrop and Crampin (2017). Considering the heterogeneity of astrocytes reported in the literature (Khakh and Sofroniew, 2015), future extensions of the model should include parameters representing the phenotypic and functional variability of the cells to obtain simulations that better reflect the complexity of astrocytic responses observed experimentally. Moreover, since Ca^2+^ puffs (irregular) and oscillations (much more regular) can be observed in the same cell for different stimulus levels, the study of Ca^2+^ dynamics offers the fascinating possibility of studying the transition from a stochastic to a deterministic regime.

It should also be pointed out that although *in vitro* models allow us to gain insight into the cellular mechanisms underlying Ca2+ regulation, they tend to simplify the cellular environment by isolating astrocytes from other cell types and their natural interactions. Indeed, as pointed out by Stogsdill et al. (2023), astrocytes are an integral part of complex neural networks, and their Ca^2+^ activity is influenced by signals from neurons and other glial cells. The use of computational models built by integrating both in vitro and *in vivo* experimental data allow a better understanding of Ca^2+^ signaling and its role in neuronal functions (Manninen et al., 2018).

## Conclusion

The article reports the findings of a comparative study of some of the most significant models for Calcium dynamics in astrocytes. The evolution from minimal models to more complex models, that consider additional biochemical processes for a more realistic description of astrocyte activity, is discussed. We compared mathematical models and experimental data of Ca^2+^ in astrocytes. The experimental data reveal complex oscillatory dynamics with different frequencies and amplitudes, reflecting the intricate regulatory mechanisms of Ca^2+^ signaling in astrocytes. Our analysis shows that the Goldbeter model, although effective in generating stable oscillations, does not allow to capture the variability in measured frequency and amplitude. The Atri model introduces a spatial wave dynamic, which could mimic some variations in the dynamics of experimental oscillations, but fails to reproduce the full range of dynamic behavior. The Li-Rinzel model, through IP_3_-dependent modulation, provides a closer approximation of the experimental data, allowing for frequency adjustments; however, it remains limited in capturing amplitude variability. The De Pittà model, on the other hand, aligns more closely with experimental observations, as it incorporates detailed GPCR and enzymatic feedback mechanisms that allow for both frequency and amplitude modulation. This latter model successfully replicates the observed changes in Ca^2+^ dynamics, making it suitable for studying the role of astrocytes in neural signaling and synaptic regulation.

It is important to note that astrocytes exhibit remarkable heterogeneity in their morphology, molecular expression, and functional responses, which varies across different brain regions and microcircuits (Khakh and Sofroniew, 2015). Incorporating this cellular diversity into computational models could enhance their ability to accurately reflect the range of astrocytic calcium dynamics observed experimentally.

Mathematical modeling of Ca^2+^ signaling in astrocytes has emerged as a critical tool for understanding the complex dynamics of the glial cells in the central nervous system. These models help elucidate the mechanisms by which astrocytes respond to neuronal activity and maintain homeostasis through Calcium signaling.

In summary, mathematical models of Ca^2+^ signaling in astrocytes are essential for deciphering the complex interactions between astrocytes and neurons. These models not only improve our understanding of normal physiological processes, but also provide a framework for studying the altered Calcium dynamics associated with various neurological disorders. Future research using these models will likely continue to reveal the intricate role of astrocytes in brain function and their potential as therapeutic targets in neurodegenerative diseases.
